# Modeling the measurement bias in interstitial glucose concentrations derived from microdialysis in skeletal muscle

**DOI:** 10.14814/phy2.15252

**Published:** 2022-04-19

**Authors:** Hugo Angleys, Leif Østergaard

**Affiliations:** ^1^ 11297 Center of Functionally Integrative Neuroscience & MINDLab Aarhus University Aarhus Denmark; ^2^ 11297 Department of Neuroradiology Aarhus University Hospital Aarhus Denmark

**Keywords:** biophysical modeling, endothelial glucose transport, interstitial glucose monitoring, microdialysis, muscle metabolism

## Abstract

Muscle tissue utilizes glucose as a fuel during exercise and stores glucose in form of glycogen during rest. The associated glucose transport includes delivery of glucose from blood plasma into the interstitial space and subsequent, GLUT‐4 facilitated diffusion into muscle cells. The extent to which the vascular endothelium acts as a barrier to glucose transport, however, remains debated. While accurate measurements of interstitial glucose concentration (IGC) are key to resolve this debate, these are also challenging as removal of interstitial fluid may perturb glucose transport and therefore bias IGC measurements. We developed a three‐compartment model to infer IGC in skeletal muscle from its local metabolism and blood flow. The model predicts that IGC remains within 5% of that of blood plasma during resting conditions but decreases more as metabolism increases. Next, we determined how microdialysis protocols affect IGC. Our model analysis suggests that microdialysis‐based IGC measurements underestimate true values. Notably, reported increases in muscle capillary permeability surface area product (PS) to glucose under the condition of elevated metabolism may owe in part to such measurements bias.

Our study demonstrates that microdialysis may be associated with significant measurement bias in the context of muscle IGC assessment. Reappraising literature data with this bias in mind, we find that muscle capillary endothelium may represent less of a barrier to glucose transport in muscle than previously believed. We discuss the impact of glucose removal on the microdialysis relative recovery and means of correcting microdialysis IGC values.

## Graphical abstract

Left panel: We developed a three‐compartment (endothelium, blood plasma, interstitium) model to infer muscle interstitial glucose concentration (IGC) from its local metabolism and blood flow. The diffusion equation is applied in each of the compartments. Right panel: Contrary to most experimental reports, the model predicts that IGC remains within 5% of that of blood plasma during resting conditions (rightmost capillaries in the outcome panel). In parallel, we predict that microdialysis‐related interstitial fluid removal creates a significant glucose concentration gradient around the microdialysis probe (right half of the outcome panel), leading to IGC measurement underestimations.

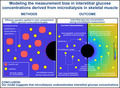

**New & Noteworthy**

Is the vascular endothelium a barrier to muscle glucose uptake? To address this debated question, we develop a three‐compartment model to infer muscle interstitial glucose concentration from its local metabolism and blood flow. Contrary to most experimental reports, interstitial glucose concentrations are predicted to remain within 5% of that of blood plasma concentrations during non‐insulin stimulated basal state. We predict that microdialysis‐related interstitial glucose removal may account for the apparent discrepancy between our predictions and experimental data.

## 1 INTRODUCTION

The uptake of glucose by skeletal muscle may increase by as much as 100‐fold during exercise compared to the resting condition (Katz et al., 1986; Richter, 2021; Richter et al., 1988; Wahren et al., 1978). Meanwhile, insulin‐mediated glucose uptake in skeletal muscle plays a key role in body glucose homeostasis. The physiological mechanisms underlying skeletal muscle glucose transport have therefore been studied extensively in both health and disease.

To be taken up by muscle cells, glucose must first be transported across the capillary endothelium, which behaves as a semi‐permeable barrier, through which glucose can diffuse. Next, glucose must be transported from the interstitial fluid into the intracellular space by means of dedicated GLUT4 transporters. Importantly, the rate at which these proteins transport glucose depends on the interstitial glucose concentration (IGC). Knowledge of IGC is therefore key to understand conditions under which glucose extraction from blood or glucose uptake by striated muscle cells, respectively, might limit glucose transport.

Muscle IGCs have been monitored extensively to study glucose transport across capillary endothelium, but reports show diverging results. Thus, some studies report IGCs close to those of blood plasma in healthy and diabetic subjects (Holmäng et al., 1998; Lönnroth et al., 1987; Moberg et al., 1997; Niklasson et al., 2000; Rosdahl et al., 1998) while others report substantially lower levels than in blood plasma (Gudbjörnsdóttir et al., 2003; MacLean et al., 1999; Maggs et al., 1995; McConell et al., 2020; Müller et al., 1995, 1996; Regittnig et al., 2003; Sandqvist et al., 2013). Taken together, reported resting interstitial glucose concentrations are in the range of 50–100% of blood plasma concentrations.

Microdialysis, which was used in most of the studies above, is regarded as a gold standard for measuring interstitial solute concentrations. Nevertheless, this method has several limitations, including the microdialysis probe's removal of the solute in question, which, in the case of glucose, is equal to approximatively $4\cdot 10^{‐11}\;\text{mol}\cdot s^{‐1}$, as inferred from probe parameter values reported in studies involving microdialysis (Müller et al., 1996), corresponding to the amount of glucose supplied by more than 500’000 capillaries to the muscle tissue at rest, or equivalently to the supply of a tissue volume larger than 90 times the volume of the probe, defined as the product between its length and its cross‐section area. This glucose removal has been hypothesized to lower solute concentrations in the surrounding tissue (Fuchi et al., 1994; Hickner et al., 1991, 1992; Lönnroth et al., 1987; Menacherry et al., 1992) and to cause solute concentrations to be underestimated (Fuchi et al., 1994). Although this concern was raised more than 25 years ago, it appears no study thus far set out to examine whether the removal of solute (notwithstanding its partial replacement via perfusate with predetermined solute concentration) indeed affects microdialysis measurements’ accuracy.

Below, we develop a framework to address this question, using a diffusion model to predict interstitial glucose concentrations as a function of tissue metabolism and blood flow. Then, we develop a second model to quantify the extent to which microdialysis affects interstitial glucose concentrations. Finally, we examine whether this measurement bias might account for discrepancies between literature IGC values, and whether the bias can be estimated and corrected for.

To address the impact of any IGC measurement bias on our conceptual understanding of blood–muscle tissue glucose transport, we employed our model to determine the apparent capillary permeability surface area product (PS) available for glucose exchange under different physiological conditions. This index is widely used to characterize glucose transport across the capillary endothelium (Gudbjörnsdóttir et al., 2003, 2005; Sandqvist et al., 2013). Notably, these studies report increased PS under conditions of increased glucose uptake, suggesting that the microvasculature somehow augments glucose extraction, for example, by *capillary recruitment*, the opening of previously closed capillaries, which has been hypothesized to explain a similar increase in PS for oxygen during exercise (Angleys & Østergaard, 2020). Specifically, we examined whether biased IGCs obtained by microdialysis might have contributed to the notion of a significant endothelial barrier to glucose blood‐tissue transport in skeletal muscle.

## 2 METHODS

### 2.1 Three‐compartment model

We developed a three‐compartment (plasma, endothelium, interstitial fluid) model to predict steady‐state glucose concentrations within each compartment as a function of glucose supply and metabolism. Accordingly, the model provides analytical expressions to calculate the glucose concentration within these compartments. The model is based on the resolution of the diffusion equation in the relevant compartments, and on the assumption that glucose is transported within the three compartments by simple diffusion, as commonly accepted in the literature (Crone & Levitt, 1984; Michel & Curry, 1999; Zierler, 1999), although in reality, glucose transport is likely to involve other processes, especially in the endothelium (Yazdani et al., 2019). We discuss the implications of this simplification in the discussion section.

The equation for plasma glucose concentration along the capillary axis can be written as:

(1) $$\begin{array}{c} Q_{a,glc}\frac{d\bar{C}\left(x\right)}{\mathrm{dx}}=‐\pi \left(r_{t}{\left(x\right)}^{2}‐r_{w}^{2}\right)\cdot M \end{array}$$

See Table 1 for a description of the model parameters.

**TABLE 1:** List of parameters used in our model

Parameter	Description	Value	Unit	Reference
α	Proportionality coefficient between $C_{i,0}‐C_{i,eff}$ and $C_{i,0}‐C_{p}$ , when steady‐state is achieved	Given by equation (18)	No unit	
$\tilde{\alpha }\left(t\right)$	Proportionality coefficient between $C_{i,0}‐C_{i,eff}\left(t\right)$ and $C_{i,0}‐C_{p}$.	Given by equation (19), $0\leq \tilde{\alpha }\left(t\right)\leq \alpha$	No unit	
β	$\frac{R\cdot f}{S_{\mathrm{probe}}}$	$6.63\cdot 10^{‐7}$ with the parameters used in the study and shown in this table	$m\cdot s^{‐1}$	
$\bar{C}\left(x\right)$	Mean plasma glucose concentration at the coordinate *x*		mM=mol/m^3^	
$C_{a}$	Arterial glucose concentration	5 during euglycemia	mM=mol/m^3^	(17, 22)
$C_{v}$	Venous glucose concentration	4.8 during euglycemia under basal conditions	mM=mol/m^3^	
$C_{i,0}$	Interstitial concentration, far from the microdialysis probe (unaffected)	4.7 at rest, given by equations (2)‐(8)	mM=mol/m^3^	
$C_{i}\left(r\right)$	Interstitial concentration at a distance *r* from the center of the probe.	Given by equation (15)	mM=mol/m^3^	
$C_{i,eff}=C_{i}\left(r_{1}\right)$	Predicted effective interstitial concentration, when employing microdialysis	Given by equation (15)	mM=mol/m^3^	
$C_{i,m}$	Predicted measured interstitial concentration, when employing microdialysis	Given by equation (25)	mM=mol/m^3^	
$C_{p}$	Perfusate glucose concentration	2	mM=mol/m^3^	(14)
$C_{\mathrm{pl}}$	Blood plasma glucose concentration		mM=mol/m^3^	
$D_{i}$	Glucose diffusion coefficient of the interstitial fluid	$9.2\cdot 10^{‐10}$	$m^{2}\cdot s^{‐1}$	(27)
$D_{p}$	Glucose diffusion coefficient in the plasma	$9.2\cdot 10^{‐10}$	$m^{2}\cdot s^{‐1}$	(27)
$D_{w}$	Glucose diffusion coefficient in the endothelium	$4.50\cdot 10^{‐14}$	$m^{2}\cdot s^{‐1}$	
*F*	Blood flow	4mL/100mL/min during rest	$m^{3}/m^{3}/s$ or mL/100mL(tissue)/min	(1, 28, 29)
*f*	Perfusate flow in the microdialysis probe	$3.33\cdot 10^{‐11}$	$m^{3}\cdot s^{‐1}$	(14)
L	Length of the microdialysis probe	$16\cdot 10^{‐3}$	m	(14)
*M*	Glucose metabolism in the tissue of skeletal muscles	0.8μmol/100mL/min in basal resting state	μmol/100mL/min or ${\text{mol/m}}^{3}/s$	(1, 28, 29)
PS	Permeability surface product	Given by equation (9)	$m^{3}/m^{3}/s=\text{mL}/\text{mL}/s$	
PS_m_	Predicted *measured* PS product, i.e., taking the influence of microdialysis on *C_i_* into account	Given by equation (31)	$m^{3}/m^{3}/s=\text{mL}/\text{mL}/s$	
$Q_{a,glc}$	Arterial glucose supply	$Q_{a,glc}=F\cdot C_{a}$	mol/s	
R	Microdialysis relative recovery	0.5	No unit	(14)
$r_{1}$	Radius of the microdialysis probe	$250\cdot 10^{‐6}$	m	(14)
$r_{p}$	Radius of the capillary plasma compartment	$2.5\cdot 10^{‐6}$	m	(30–32)
$r_{t,a}$	Radius of the tissue cone assumed by our model, arterial side.	$28\cdot 10^{‐6}$	m	
$r_{t,v}$	Radius of the tissue cone assumed by our model, venous side.	$22\cdot 10^{‐6}$	m	
$r_{w}$	Capillary outer radius (plasma + endothelial wall)	$3.1\cdot 10^{‐6}$	m	(30–32)
$S_{\mathrm{probe}}$	Microdialysis probe exchange surface	$2\pi r_{1}L$	$m^{2}$	(14)
$V_{i}$	Relative interstitial volume in the tissue	20%	No unit	(33–36)

Integrating Equation (1) on a cone shaped domain yields:

(2) $$\begin{array}{c} \bar{C}\left(x\right)=C_{a}‐\frac{\pi xM}{Q_{a,glc}}\left\{r_{t,a}^{2}\left[1+\frac{x}{L}\left(\frac{r_{t.v}}{r_{t.a}}‐1\right)+\frac{1}{3}{\left(\frac{x}{L}\right)}^{2}{\left(\frac{r_{t,v}}{r_{t,a}}+1\right)}^{2}\right]‐r_{w}^{2}\right\} \end{array}$$

In the plasma the diffusion equation can be written:

(3) $$D_{p}\frac{1}{r}\frac{d}{\mathrm{dr}}\left(r\frac{\mathrm{dC}}{\mathrm{dr}}\right)=‐M\cdot \frac{r_{t}{\left(x\right)}^{2}‐r_{w}^{2}}{r_{p}^{2}}$$

In the endothelium it can be written:

(4) $$D_{w}\frac{1}{r}\frac{d}{\mathrm{dr}}\left(r\frac{\mathrm{dC}}{\mathrm{dr}}\right)=0$$

While in the interstitial fluid, it can be written:

(5) $$D_{i}\frac{1}{r}\frac{d}{\mathrm{dr}}\left(r\frac{\mathrm{dC}}{\mathrm{dr}}\right)=\frac{M}{V_{i}}$$

Solving these equations, the concentration $C(r_{P})$ is found to be equal to

(6) $$\begin{array}{c} C\left(r_{p},x\right)=\bar{C}\left(x\right)‐\frac{M\left(r_{t}{\left(x\right)}^{2}‐r_{w}^{2}\right)}{8D_{p}} \end{array}$$

The concentration gradient across the endothelium is equal to

(7) $$C\left(r_{p},x\right)‐C\left(r_{w},x\right)=M\cdot \left(r_{t}{\left(x\right)}^{2}‐r_{w}^{2}\right)\cdot \frac{\ln\left(\frac{r_{w}}{r_{p}}\right)}{2D_{w}}$$

Note the important result of Equation (7), which predicts that the cross‐endothelial glucose gradient is proportional to its metabolism.

The concentration within the interstitial fluid is found to be equal to:

(8) $$C\left(r_{w}\leq r\leq r_{t},x\right)=C\left(r_{w},x\right)‐\frac{M}{4\cdot V_{i}\cdot D_{i}}\left(r_{w}^{2}‐r^{2}+2r_{t}{\left(x\right)}^{2}\cdot \ln\left(\frac{r}{r_{w}}\right)\right)$$

### 2.2 Calibration of glucose diffusion coefficients in the different compartments

The diffusion coefficients in the plasma and in the extracellular fluid are set to $9.2\cdot 10^{‐10}\;m^{2}\cdot s^{‐1}$, as reported in the literature (Mignot & Junter, 1990).

The endothelial wall's diffusion coefficient *D*_w_ used in our model can be inferred from the time required for interstitial glucose concentration to reach equilibrium after a step increase in glucose plasma concentration. Accordingly, Regittnig and colleagues determined experimentally that it takes about 15 min for interstitial fluid glucose tracer concentrations to reach equilibrium after tracer infusion in the vasculature (Regittnig et al., 2003). To determine the equilibrium time given the parameters used in our model, we solved the time‐dependent diffusion equation in the endothelium and interstitial compartments using the Partial Differential Equation (PDE) Toolbox in Matlab. Accordingly, we set the endothelial diffusion coefficient to $4.50\cdot 10^{‐14}\;m^{2}\cdot s^{‐1}$, which yields a similar equilibrium time as measured in Regittnig et al. (2003).

### 2.3 Application to the model: Computation of the PS product

Knowing the glucose concentration in the interstitial compartment, the equivalent endothelial PS product can be calculated as:

(9) $$\text{PS}=‐F\cdot \ln\left(\frac{C_{v}‐C_{i}}{C_{a}‐C_{i}}\right)$$

### 2.4 Modeling glucose removal by a microdialysis probe

Glucose removal from the interstitial fluid by microdialysis imposes an artificial steady‐state condition on the compartmental system described above, altering glucose concentrations within the interstitial fluid, across the capillary endothelium, and in plasma in a manner that depends on the probe glucose removal rate. Specifically, interstitial glucose removal leads to increased glucose extraction from capillary blood and hence to a fall in the plasma glucose concentration, $\Delta C_{\mathrm{pl}}$. Moreover, the higher glucose flux across the endothelium leads to an increase in the trans‐endothelial glucose gradient, $\Delta G_{e}$. Below, we quantify the resulting interstitial glucose concentration drop $\Delta C_{i}=\Delta C_{\mathrm{pl}}+\Delta G_{e}$. Note that $\Delta C_{\mathrm{pl}}$, $\Delta G_{e}$, and $\Delta C_{i}$ vary with the distance to the probe, and tend to zero as this distance increases.

We define $\Delta C_{i}\left(r\right)<0$ to be the difference between $C_{i}\left(r\right)$, the effective interstitial glucose concentration at a distance *r* from the center of the probe, and $C_{i,0}$, the interstitial glucose concentration in tissue unaffected by the glucose removal, for example, in a tissue area far away from the probe.

To quantify $\Delta C_{i}$ in terms of known parameters, we first write the diffusion equation in the interstitial compartment. Considering that capillary dimensions are small compared to the tissue microdialysis probe, the diffusion equation in the tissue can be written:

(10) $$D_{i}\cdot V_{i}\cdot \frac{1}{r}\frac{d}{\mathrm{dr}}\left(r\frac{dC_{i}}{\mathrm{dr}}\right)=‐\gamma \left(r\right)$$

with γ>0 being the rate at which the glucose removed by the probe is supplied by capillaries, per unit volume of muscle tissue, and *r* the distance from the center of the microdialysis probe. Note that Equation (10) corresponds to steady‐state concentrations. Time dependent solutions can be considered by including the time derivative of $C_{i}$ in the right hand term of Equation (10), see paragraph *Determining the characteristic time to reach steady*‐*state IGC*, in the *Results* section.

Assuming that glucose uptake is uniformly distributed along the capillary length, the resulting drop in glucose plasma concentration is equal in average to:

(11) $$\Delta C_{\mathrm{pl}}\left(r\right)=‐C_{a}\cdot \frac{\Delta E\left(r\right)}{2}$$

where ΔE is the extra glucose extraction fraction resulting from probe glucose removal. ΔE can be expressed as a function of $\gamma \left(r\right)$ as $\frac{\gamma \left(r\right)}{F\cdot C_{a}}$, with γ>0. $\Delta C_{\mathrm{pl}}$ can in turn be explicitly expressed as a function of $\gamma \left(r\right)$ as:

(12) $$\Delta C_{\mathrm{pl}}\left(r\right)=\frac{‐\gamma \left(r\right)}{2F}$$

Equation (7) allows us to express the additional glucose concentration gradient across the endothelium $\Delta G_{e}$ in terms of $\gamma \left(r\right)$ as:

(13) $$\Delta G_{e}\left(r\right)=‐\gamma \left(r\right)\cdot \left(r_{t}^{2}‐r_{w}^{2}\right)\cdot \frac{\ln\left(\frac{r_{w}}{r_{p}}\right)}{2D_{w}}$$

In total, the extra drop in the interstitial fluid due to the glucose removed by the microdialysis probe is equal to:

(14) $$\Delta C_{i}\left(r\right)=\Delta C_{\mathrm{pl}}\left(r\right)+\Delta G_{e}\left(r\right)=‐\gamma \left(r\right)\cdot \left(\frac{1}{2F}+\left(r_{t}^{2}‐r_{w}^{2}\right)\cdot \frac{\ln\left(\frac{r_{w}}{r_{p}}\right)}{2D_{w}}\right)$$

Equation (10) can thus be rewritten in terms of known parameters as:

(15) $$D_{i}\cdot V_{i}\cdot \frac{1}{r}\frac{d}{\mathrm{dr}}\left(r\frac{dC_{i}\left(r\right)}{\mathrm{dr}}\right)=\frac{C_{i}\left(r\right)‐C_{i,0}}{\frac{1}{2F}+\frac{\left(r_{t}^{2}‐r_{w}^{2}\right)}{2D_{w}}\cdot \ln\left(\frac{r_{w}}{r_{p}}\right)}$$

#### 2.4.1 Boundary conditions

The glucose flux *j* into the probe can be expressed in terms of the probe parameters as probe's removal rate $R\cdot f\cdot \left(C_{i}\left(r_{1}\right)‐C_{p}\right)$, divided by its surface area, $S_{\mathrm{probe}}\;:\;j\left(r_{1}\right)=\frac{R\cdot f}{S_{\mathrm{probe}}}\left(C_{i}\left(r_{1}\right)‐C_{p}\right)$, with the values of the different parameters as shown in Table 1.

Using Fick's law of diffusion, $j=‐D_{i}\cdot V_{i}\cdot \frac{dC_{i}}{\mathrm{dr}}$, we can express the boundary conditions that Equation (15) must fulfill as:

(16) $$\left\{\begin{array}{cc} {\left.\frac{dC_{i}}{\mathrm{dr}}\right|}_{r=r_{1}}=‐\frac{R\cdot f}{D_{i}\cdot V_{i}\cdot S_{\text{probe}}}\cdot \left(C_{i}\left(r_{1}\right)‐C_{p}\right) & \left(a\right)\\ C_{i}\left(r_{2}\right)=C_{i,0} & \left(b\right) \end{array}\right.$$

With $r_{2}\gg r_{1}$ being a distance far enough from the probe for tissue glucose levels to be unaffected by probe glucose removal. We solved Equation (15) with boundary conditions (16) numerically using PDE toolbox in Matlab.

It can be shown, as a consequence of the linearity of the Laplacian and of the derivation involved in Equations (15) and (16), that the concentration *C_i_*, solution of Equation (15) with boundary conditions (Sandqvist et al., 2013), is such that for any fixed *r_0_* equal to or larger than *r_1_*, $C_{i}\left(r_{0}\right)‐C_{p}$, and in turn $C_{i,0}‐C_{i}\left(r_{0}\right)$, are proportional to $C_{i,0}‐C_{p}$. In particular, for $r_{0}=r_{1}:$

(17) $$C_{i,0}‐C_{i,eff}\propto C_{i,0}‐C_{p}$$

With $C_{i,eff}\equiv C_{i}\left(r_{1}\right)$ being the *effective* concentration at the probe's immediate vicinity. Accordingly, we refer to this concentration in the following as $C_{i,eff}$. Note the importance of relation (17), which means that changes in $C_{i,eff}$ can be directly inferred from a change in $C_{i,0}$, $C_{p}$, or both. Accordingly, Equation (15) only needs to be solved once to determine $C_{i,eff}$ for any arbitrary value of $C_{i,0}$ or $C_{p}$.

In the following, $\alpha \in [0,1[$ denotes the proportionality coefficient such that $C_{i,0}‐C_{i,eff}=\alpha \cdot \left(C_{i,0}‐C_{p}\right)$. Alternatively, $C_{i,eff}$ can be written as:

(18) $$C_{i,eff}=C_{i,0}\cdot \left(1‐\alpha \right)+C_{p}\cdot \alpha$$

that is, $C_{i,eff}$ is the weighted mean between $C_{i,0}$ and $C_{p}$, where α can be interpreted as a measure of the bias amplitude.

In the more general case in which steady‐state is not achieved, relation (17) still holds, and the proportionality coefficient depends on time: $C_{i,0}‐C_{i,eff}\left(t\right)=\tilde{\alpha }\left(t\right)\cdot \left(C_{i,0}‐C_{p}\right)$, which can be rewritten as:

(19) $$C_{i,eff}\left(t\right)=C_{i,0}\cdot \left(1‐\tilde{\alpha }\left(t\right)\right)+C_{p}\cdot \tilde{\alpha }\left(t\right)$$

with *t* denoting the time after microdialysis onset and $\tilde{\alpha }\left(t\right)\in \left[0,\alpha \right]$ being a monotonically increasing function of time, see paragraph *Determining the characteristic time to reach steady*‐*state* in the *Results* section.

### 2.5 Bias in the relative recovery coefficient, and propagation in the measure estimate

IGC measurements performed with microdialysis are inferred indirectly from dialysate concentration and from the relative recovery, a parameter that we note *R* in the following, according to the relation: $C_{i}=\frac{C_{d}‐\left(1‐R\right)\cdot C_{p}}{R}$, with $C_{d}$ being the dialysate (=perfusate after its transit in the microdialysis probe) concentration. The recovery is related to the probe's apparent permeability and is equal to

(20) $$R\equiv \frac{C_{d}‐C_{p}}{C_{i}‐C_{p}}$$

with $C_{i}$ being the effective interstitial concentration at the vicinity of the probe. Accordingly, any bias or imprecision in the determined value of *R* would lead to a biased measured IGC. We call $R_{m}$ the *measured* recovery, determined experimentally, and $C_{i,m}$, the *measured* IGC, inferred experimentally from $R_{m}$ and from $C_{d}$:

(21) $$C_{i,m}=\frac{C_{d}‐\left(1‐R_{m}\right)\cdot C_{p}}{R_{m}}$$

That is, $C_{i,m}$ combines two sources of imprecision (bias): (i) the first contribution comes from $C_{d}$, that may be biased because of glucose removal, and (ii) the second contribution is related to $R_{m}$, that may have been calibrated inaccurately. In the following paragraphs, we examine the extent to which glucose removal biases the determined value of *R* when employing the no‐net‐flux and the internal reference calibration techniques. The value of $R_{m}$ and $C_{i,m}$ is then expressed in terms of known parameters.

#### 2.5.1 No‐net‐flux calibration

The no‐net‐flux calibration technique consists in measuring the dialysate concentration $C_{d}$ for different perfusate concentration values $C_{p}$. Indeed, rewriting Equation (20), $C_{d}$ can be expressed as a function of $C_{p}$, $C_{i}$, and *R* as: $C_{d}=\left(1‐R\right)\cdot C_{p}+R\cdot C_{i}$. Thus, $C_{d}$ varies linearly with $C_{p}$, with a slope coefficient (1−*R*), from which the relative recovery is inferred in practice by linear regression.

$C_{d}$ can be written as a function of $C_{p}$ and $C_{i,eff}$ as:

(22) $$C_{d}=\left(1‐R_{0}\right)\cdot C_{p}+R_{0}\cdot C_{i,eff}\left(t;C_{p}\right)$$

where $C_{i,eff}$ is a function of $C_{p}$ (Equation 19), and $R_{0}\equiv \frac{C_{d}‐C_{p}}{C_{i,eff}‐C_{p}}$ is the true, unbiased recovery value, for a given set of probe parameters and perfusate flow value. Equation can be rewritten by expressing $C_{i,eff}$ as a function of $C_{i,0}$ and $C_{p}$ (Equation 19):

(23) $$C_{d}=\left(1‐R_{0}\left(1‐\tilde{\alpha }\left(t\right)\right)\right)\cdot C_{p}+R_{0}\left(1‐\tilde{\alpha }\left(t\right)\right)\cdot C_{i,0}$$

Note the importance of this result, which explains the observed linear relationship between $C_{d}$ and $C_{p}$ even when glucose is removed by the microdialysis probe, although this linearity used to seem incompatible with glucose removal by the probe (Fuchi et al., 1994). The estimated measured coefficient $R_{m}$ from the calibration is thus:

(24) $$R_{m}=\left(1‐\tilde{\alpha }\left(t\right)\right)\cdot R_{0}$$

Equation (21) can be rewritten as a function of $C_{p}$, $C_{i,0}$, α and $\tilde{\alpha }$ by reinjecting Equation (23), for which steady‐state is assumed, and (24) in (21), as:

(25) $$C_{i,m}=\frac{1‐\alpha }{1‐\tilde{\alpha }}\cdot C_{i,0}+\frac{\alpha ‐\tilde{\alpha }}{1‐\tilde{\alpha }}\cdot C_{p}$$

$C_{i,m}$ is thus a weighted mean between $C_{p}$ and $C_{i,0}$. Accordingly, if steady‐state is completely achieved before each measurement of $C_{d}$ during the calibration procedure, $\tilde{\alpha }\left(t\right)=\alpha$, and $C_{i,m}=C_{i,0}$. If on the contrary, $\tilde{\alpha }=0$, and $R_{m}=R_{0}$ is unbiased, then $C_{i,m}=\left(1‐\alpha \right)\cdot C_{i,0}+\alpha \cdot C_{p}=C_{i,eff}$. These predictions are discussed in more details below in the Discussion.

#### 2.5.2 Internal reference calibration

The internal reference technique consists in determining the extraction fraction of a glucose tracer contained in the perfusate. While the tracer concentration in the interstitium is assumed to be zero, in reality, the tracer accumulates in the interstitium around the probe, and therefore affects net diffusion of tracer outside of the probe.

Accordingly, it can be shown that the measured recovery is biased by a factor $1‐\frac{C_{i}^{*}}{C_{p}^{*}}$ when $C_{p}^{*}\ll C$, with $C_{i}^{*}$ and $C_{p}^{*}$ being the glucose tracer concentrations in the interstitial fluid and in the perfusate, respectively. The measured relative recovery is thus:

(26) $$R_{m}=\left(1‐\frac{C_{i}^{*}}{C_{p}^{*}}\right)\cdot R_{0}$$

We made a model to determine the extent to which the tracer accumulates at the vicinity of the probe, as a function of $C_{p}^{*}$. The tracer follows same equations as native glucose. The diffusion equation for the glucose tracer in the interstitial fluid can be written:

(27) $$D_{i}\cdot V_{i}\cdot \frac{1}{r}\frac{d}{\mathrm{dr}}\left(r\frac{dC_{i}^{*}\left(r\right)}{\mathrm{dr}}\right)=\frac{C_{i}^{*}\left(r\right)}{\frac{1}{2F}+\frac{\left(r_{t}^{2}‐r_{w}^{2}\right)}{2D_{w}}\cdot \ln\left(\frac{r_{w}}{r_{p}}\right)}+\frac{M\cdot C_{i}^{*}\left(r\right)}{C_{i}\left(r\right)}$$

with boundary conditions:

(28) $$\left\{\begin{array}{cc} {\left.\frac{dC_{i}^{*}}{\mathrm{dr}}\right|}_{r=r_{1}}=‐\frac{R\cdot f}{D_{i}\cdot V_{i}\cdot S_{\text{probe}}}\cdot \left(C_{i}^{*}\left(r_{1}\right)‐C_{p}^{*}\right) & \left(a\right)\\ C_{i}^{*}\left(r_{2}\right)=0 & \left(b\right) \end{array}\right.$$

In Equation (28), the terms $\frac{C_{i}^{*}\left(r\right)}{\frac{1}{2F}+\frac{\left(r_{t}^{2}‐r_{w}^{2}\right)}{2D_{w}}\cdot \ln\left(\frac{r_{w}}{r_{p}}\right)}$ and $\frac{M\cdot C_{i}^{*}\left(r\right)}{C_{i}\left(r\right)}$, correspond to the tracer removed from the tissue via capillaries, and metabolized, respectively. Numerically, the contribution of the latter term is negligible compared to the first one. This system of equations is therefore almost similar to the system solved above (15)–(16). In particular, the quantity $\frac{C_{i}^{*}\left(t\right)}{C_{p}^{*}}$ is almost equal to $\tilde{\alpha }\left(t\right)\equiv \frac{C_{i,0}‐C_{i,eff}\left(t\right)}{C_{i,0}‐C_{p}}$ introduced above, meaning that the estimated measured coefficient $R_{m}$ from the internal reference calibration technique is biased in the same way as in the no‐net‐flux calibration technique. In turn, the measured IGC $C_{i,m}$ can be written with the same expression (25) when using both calibration techniques.

## 3 RESULTS

### 3.1 Glucose concentration gradients within the interstitial fluid

The analytical expression for the glucose concentration gradient within the interstitial fluid compartment can be derived analytically from Equation (8) as:

(29) $$\nabla C_{i}\left(r\right)=\frac{M}{2\cdot V_{i}\cdot D_{i}}\cdot \left(r‐\frac{r_{t}^{2}}{r}\right)$$

Due to the high value of the diffusion coefficient *D_i_*, the gradient within the interstitial fluid is very low compared to the glucose concentrations in the plasma. The glucose concentration under resting conditions 25 µm away from the capillary is predicted to be only $4.8\cdot 10^{‐4}$ mM lower, that is, approximately 0.01% lower than in the immediate vicinity of the capillary. Variations in glucose concentration within the interstitial compartment are therefore predicted to be insignificant. In fact, even under conditions of elevated metabolism, glucose concentration within the interstitial compartment are predicted to vary by less than $5\cdot 10^{‐2}$ mM.

### 3.2 Glucose concentration gradients across the endothelium

Equation (7) gives the analytical expression for the glucose concentration gradient across the endothelium. This corresponds to a concentration difference at rest of 0.2 mM across the endothelium, that is, approximatively 4% of the normal plasma glucose concentration and of the same order of magnitude as the arteriovenous glucose concentration difference during rest. Under condition of hyperinsulinemia, due to the significant increase in metabolism – typically 15‐fold increase or more, the concentration difference across the endothelium is predicted to increase to values equal to 3.0–3.5 mM.

### 3.3 Effects of microdialysis on glucose interstitial concentrations

**FIGURE 1:**
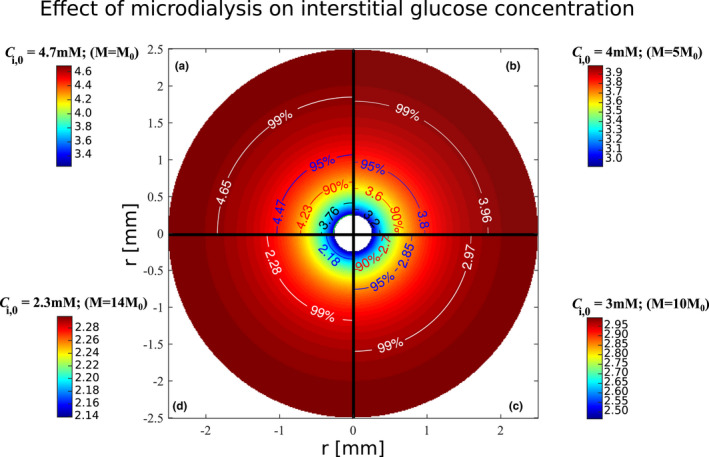
Illustration of the effects of glucose removal by the microdialysis probe on interstitial glucose concentrations. The figure shows the predicted interstitial concentration distribution within the interstitial compartment in a cross section with the microdialysis probe in the center. The probe's cross section is represented at the center of the figure as a white disk (radius = 0.25 mm). The figure is divided in four quarters, each of them corresponding to a different assumed interstitial concentration $C_{i,0}$. Isocontours’ labels show the concentration (in mM) and the corresponding $C_{i,0}$ fraction (in percentage) it represents. The isocontours show concentration levels equal to 99%, 95%, 90% 80%, and 70%, respectively, of $C_{i,0}$, which are represented in white, blue, red, black, and green, respectively. Similar vascular glucose supply is assumed in the figures’ four quarters. Blood flow is assumed to be equal to 4 ml/100 ml/min, and arterial glucose concentration is assumed to be equal to 5 mM. *R*, *f* and $S_{\text{probe}}$ are set to values as shown in Table 1. In parentheses is indicated for each quarter the glucose metabolism value corresponding to $C_{i,0}$, where *M*_0_ is the resting glucose metabolism equal to 0.8 μmol/100 mol/min. $C_{i,0}$: Interstitial concentration far enough from the probe not to be affected

**FIGURE 2:**
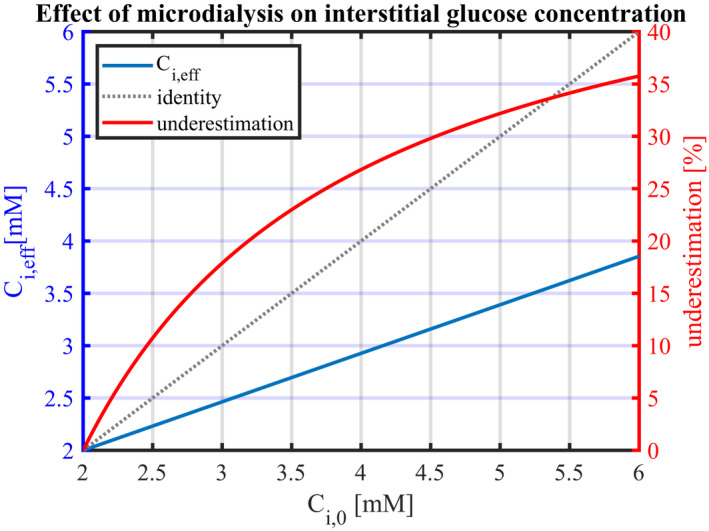
Predicted measured interstitial glucose concentration $C_{i,eff}$ in the vicinity of the microdialysis probe as a function of the ‘true’ interstitial concentration $C_{i,0}$ (blue line). The corresponding relative underestimation is shown as a red line. The perfusate concentration in the microdialysis probe $C_{p}$ is assumed to be 2 mM. Blood flow is assumed to be 4 ml/100 ml/min, and arterial glucose concentration 5 mM. *R*, *f* and $S_{\text{probe}}$ are set to values as shown in Table 1. Reduction in $C_{i,0}$ can be the result of an increase in plasma insulin concentration and, in turn, in glucose uptake. $C_{i,eff}$ **:** effective interstitial glucose concentration at the probe's immediate vicinity when employing microdialysis; $C_{i,0}$: Interstitial concentration far enough from the probe for it not to be affected

**FIGURE 3:**
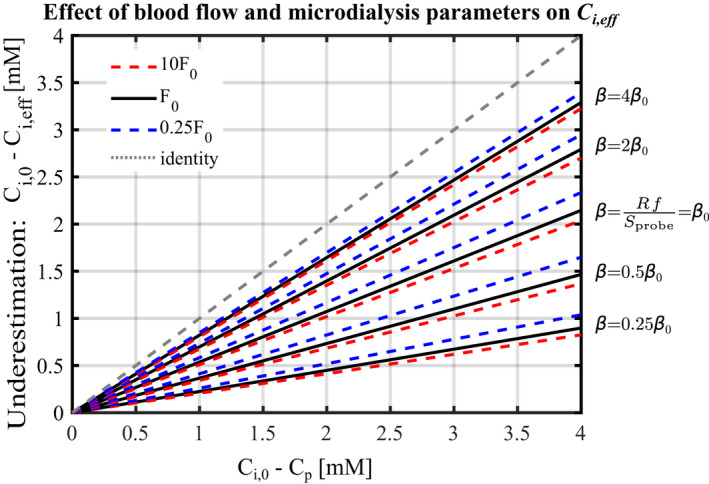
Measured concentration difference $C_{i,eff}‐C_{p}$ as a function of the ‘true’ concentration difference $C_{i,0}‐C_{p}$ for different blood flow and $\beta =\frac{R\cdot f}{S_{\text{probe}}}$ values. Black solid lines, dashed red lines and dashed blue lines show predictions for *F* = *F*_0_ = 4 ml/100 ml/min, *F* = 40 ml/100 ml/min, and *F* = 1 ml/100 ml/min, respectively. Predictions for different β values in the range $\left[0.25\beta_{0};4\beta_{0}\right]$ have been used as indicated in the figure, with $\beta_{0}$ corresponding to parameters values shown in Table 1, and used in Figures 1 and 2. The grey dotted line shows the identity line, corresponding to the relation that would be observed between *C*_i,eff_ and *C*_i.0_ if microdialysis were unbiased. $C_{i,eff}$ **:** Effective interstitial glucose concentration at the probe's immediate vicinity when employing microdialysis; *C*_i,0_: interstitial concentration far enough from the probe not to be affected

Figures 1 and 2 illustrate the effects of the microdialysis probe on the interstitial glucose concentration. Figure 1 shows the predicted distribution of glucose concentration within the interstitial compartment. The four quarters in Figure 1 show predictions for different interstitial concentrations $C_{i,0}$. As interstitial concentrations are predicted to depend on the cellular metabolism (see Equation 7), higher $C_{i,0}$ values (quarter A) reflects resting metabolism and basal plasma insulin concentrations, while quarters B, C, and D correspond to conditions of higher metabolism, for example as a result of increased insulin plasma concentrations. Blood flow is assumed to remain constant across all figures’ panels, while bearing in mind that insulin is weakly vasodilating. This choice was made to illustrate how solutions to Equations (15)–(16) vary with a single parameter $C_{i,0}$, with all other parameters being fixed. The influence of *F* on IGC is illustrated further below in Figure 3.

Figure 1 shows how glucose removal by the microdialysis probe is predicted to reduce the interstitial glucose concentration within a cylindrical region of radius equal to 1–2 mm. Concentrations further away from the probe are predicted to be affected by less than 1–5%. The tissue volume, in which the probe substantially affects interstitial concentrations, is predicted to correlate positively with the initial concentration $C_{i,0}$. The figure's isocontours, which correspond to certain fractions of $C_{i,0}$, are represented by identical colors across the figure's quarters. By following a given isocontour color across the figure quarters, the relation between $C_{i,0}$ and microdialysis effect on the interstitial concentration can therefore be appreciated: The higher $C_{i,0}$ is, the larger the diameter of a given isocontour, meaning that the tissue volume in which interstitial glucose concentrations are affected by the sampling, is larger. Furthermore, Figure 1 shows that at a given distance from the microdialysis probe, the extent to which interstitial concentrations are affected correlates with $C_{i,0}$ values. This is further illustrated in Figure 2, which shows predictions for $C_{i,eff}=C_{i}\left(r_{1}\right)$, the concentration in the probe's immediate vicinity, as a function of the interstitial concentration $C_{i,0}$. Figure 2 also shows the resulting, relative underestimation of “true” interstitial glucose concentration, which increases from 0% when $C_{i,0}=C_{p}=2.0\;\text{mM}$ to 36% for $C_{i,0}=6.0\;\text{mM}$, corresponding to an absolute concentration difference between $C_{i,0}$ and $C_{i,eff}$ of 2.1mM. For $C_{i,0}=4.7\;\text{mM}$, which is the assumed interstitial concentration under resting conditions, the measured glucose concentration is 3.3 mM, corresponding to a relative underestimation of 31%, and to a glucose removal rate of $2.41\cdot 10^{‐11}\;\text{mol}\cdot s^{‐1}$.

Proportionality relationship (17) is illustrated by the representation of $C_{i,eff}$ as a function of $C_{i,0}$ in Figure 2, as a straight line passing through the point with coordinates ( $C_{p}$, $C_{p}$). The slope of the line in Figure 2 is determined by the terms $\frac{1}{2F}+\frac{\left(r_{t}^{2}‐r_{w}^{2}\right)}{2D_{w}}\cdot \ln\left(\frac{r_{w}}{r_{p}}\right)$ and $\beta \equiv \frac{R\cdot f}{S_{\mathrm{probe}}}$, which appear in Equations (15) and (16), respectively. Accordingly, the concentration *C_i_* does not depend on parameters *R*, *f*, *S*_probe_ independently: A given variation in *R* will have the same effect on $C_{i,eff}$ as the same relative variation in *f*.

Figure 3 illustrates the extent to which probe parameters and blood flow influence the microdialysis measurement bias. The figure shows the linear relationship between the concentration differences $C_{i,0}‐C_{i,eff}$ and $C_{i,0}‐C_{p}$ for different values of *F* and parameter $\beta =\frac{R\cdot f}{S_{\mathrm{probe}}}$, in the range 25–400% of the values that have been used in Figure 2. This figure allows for direct reading of the model predictions as a function of perfusate glucose concentrations. For instance, with probes parameters set as in Figure 1a, with $C_{i,0}=4.7\;\text{mM}$ and $C_{p}=2\;\text{mM}$, that is $C_{i,0}‐C_{p}=2.7\;\text{mM}$, the underestimation $C_{i,0}‐C_{i,eff}$ is expected to be equal to 1.4mM, that is, $C_{i,eff}=3.3\;\text{mM}$. As a result of the linear relation between the underestimation $C_{\text{i,0}}‐C_{i,eff}$ and $C_{i,0}‐C_{p}$, respectively, increasing $C_{p}$ to 3.3mM, that is, reducing the difference $C_{i,0}‐C_{p}$ by half to 1.35 mM, would reduce the predicted underestimation $C_{i,0}‐C_{i,eff}$ by half to 0.7 mM, yielding $C_{i,eff}=4\;\text{mM}$.

The figure shows that line slopes vary to a greater extent with β than it does with *F*, which illustrates that IGCs are more sensitive to variations in probe parameters than in blood flow. When probe parameter *R* or *f* increases, the slope increases, meaning that the underestimation made by microdialysis increases. Indeed, higher *R* or *f* corresponds to higher glucose flow rate removed by the microdialysis probe, all other parameters being equal, and therefore to higher glucose flux into the probe. When *F* increases, vascular glucose supply increases, and capillaries contribute to a greater extent to replenish the glucose removed by the probe. As a consequence, the probe affects interstitial concentrations in a smaller volume, and the overall influence of the probe is therefore lower. In Figure 3, this corresponds to a lower slope, and lower underestimation. Figure 3 allows us to evaluate the influence of a combined increase in *F* and decrease in $C_{i,0}$, as observed for instance under conditions of hyperinsulinemia. While increased *F* and decreased $C_{i,0}$ both reduce IGC underestimation, the influence of *F* is predicted to be negligible compared to that of $C_{i,0}$.

A quick overview of the model's predictions can be obtained without solving Equation (15) explicitly. Instead, predictions may be inferred directly from Figure 3, which presents the relationship between $C_{i,eff}$ and $C_{i,0}‐C_{p}$ for β values in the range $1.66\cdot 10^{‐7}‐2.65\cdot 10^{‐6}\;m\cdot s^{‐1}$. A quick $C_{i,0}$ estimate can in turn be inferred by assuming that glucose uptake is uniformly distributed along the capillary length, and by neglecting solute concentrations variations within the interstitium, simplifying $C_{i,0}$ expression to:

(30) $$C_{i,0}=\frac{C_{a}+C_{v}}{2}‐M\cdot \left(r_{t}^{2}‐r_{w}^{2}\right)\cdot \frac{\ln\left(\frac{r_{w}}{r_{p}}\right)}{2D_{w}}$$

### 3.4 Comparison with experimental data

**FIGURE 4:**
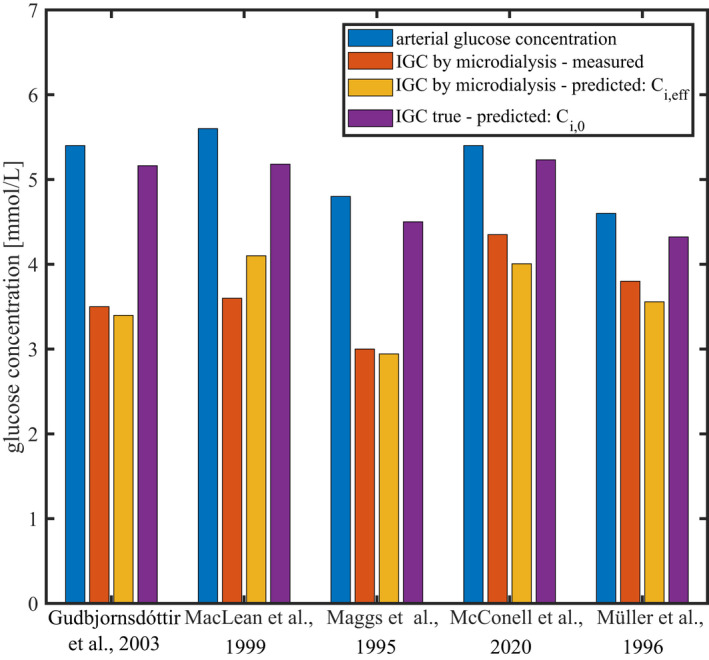
Comparison between our model predictions and experimental measurements under euglycemic resting conditions, reported in different studies, as indicated in the figure. Blue bars show reported arterial glucose concentrations. For each condition, predictions have been made assuming microdialysis probe parameters, blood flow, and tissue metabolism as reported in the different studies

**FIGURE 5:**
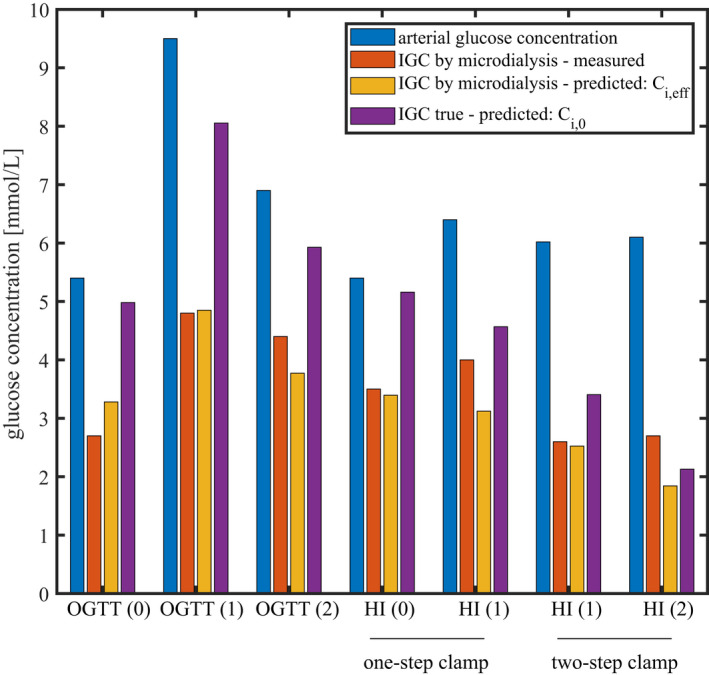
Comparison between our model predictions and experimental measurements in different physiological conditions reported in Gudbjörnsdóttir et al. (2003). Blue and red bars show arterial glucose concentrations and interstitial glucose concentrations measured by microdialysis, respectively. Yellow bars show microdialysis derived IGC predicted by our model while purple bars show the ‘true’ IGC predicted by our model, assuming that the microdialysis probe does not affect interstitial glucose concentrations. Predictions in this figure have been made assuming probe parameters as reported in Gudbjörnsdóttir et al. (2003) recovery rate *R* = 0.3; perfusate flow $f=4.17\cdot 10^{‐11}m^{3}\cdot s^{‐1}$; $C_{p}=1.5\;\text{mM}$; other parameters as shown in Table 1. Abbreviations: OGTT: oral glucose tolerance test; HI: hyperinsulinemia. Conditions‘(0)’ refer to baseline

We used the models above to predict which interstitial glucose concentrations microdialysis experiments would arrive at under different physiological conditions – See Figures 4 and 5 and Tables 2 and 3 for corresponding values. Figures 4 and 5 compare our model predictions with experimental data (red bars). These measurements are markedly lower than our predictions (purple bars). To examine whether these discrepancies might be attributable to a bias caused by microdialysis probe glucose removal, we used our second model to infer the concentrations $C_{i,eff}$ that would be *measured* by the microdialysis, from the predicted interstitial concentrations $C_{i,0}$. The corresponding predicted measurements appear as yellow bars in Figures 4 and 5.

**TABLE 2:** Glucose concentrations values shown in Figure 4

Study	Arterial glucose concentration (measured) (mM)	Measured IGC (microdialysis) (mM)	Predicted effective IGC, $C_{i,eff}$ (microdialysis) (mM)	Relative difference with measurements (%)	Predicted IGC (true, $C_{i,0}$) (mM)	Relative difference with measurements (%)
Gudbjörnsdóttir et al. (2003)	5.4	3.5	3.4	−2.9	5.2	+48
MacLean et al. (1999)	5.6	3.6	4.1	+14	5.2	+44
Maggs et al. (1995)	4.8	3.0	2.9	−1.9	4.5	+50
McConell et al. (2020)	5.4	4.4	4.0	−7.9	5.2	+20
Müller et al. (1996)	4.6	3.8	3.6	−6.3	4.3	+14
**Mean (of absolute values)**				**6.6**		**35**

**TABLE 3:** Glucose concentrations values and PS product measured by Gudbjörnsdóttir et al. (2003), and predicted by our model. Predictions in this table have been made assuming probe parameters as reported in Gudbjörnsdóttir et al. (2003): recovery rate *R* = 0.3; perfusate flow $f=4.17\cdot 10^{‐11}\;m^{3}\cdot s^{‐1}$; $C_{p}=1.5\;\text{mM}$; other parameters as shown in Table 1. OGTT(0) and HI(0) denote basal states. OGTT: oral glucose tolerance test; HI: hyperinsulinemia; IGC: interstitial glucose concentration; PS: endothelial permeability‐surface product for glucose exchange. *In these conditions, the PS product could not be determined by our model due to the elevated extraction fraction values

Condition	Arterial glucose concentration (measured) (mM)	IGC (mM)					PS product $\times 10^{‐4}\text{mL/mL/s}$		
		Measured (microdialysis)	Predicted –Microdialysis $C_{i,eff}$	Relative difference with measurements (%)	Predicted ‐ true $C_{i,0}$	Relative difference with measurements (%)	Measured	PS_m_ Predicted –Microdialysis, based on *C_i_*_,_ *_eff_*	Predicted – true, based on C*_i_*_,_ *_0_*
OGTT(0)	5.4	2.7	3.3	+21	5.0	+85	0.48	0.69	NA*
OGTT(1)	9.5	4.8	4.9	+1	8.1	+68	0.95	1.2	NA*
OGTT(2)	6.9	4.4	3.8	−14	5.9	+35	1.1	1.2	NA*
HI(0)‐one‐step	5.4	3.5	3.4	−3	5.2	+47	0.33	0.44	11
HI(1)‐one‐step	6.4	4.0	3.1	−22	4.6	+14	3.8	2.9	7.7
HI(1)‐two‐step	6.0	2.6	2.5	−3	3.4	+31	3.7	4.5	7.0
HI(2)‐two‐step	6.1	2.7	1.8	−32	2.1	−21	7.5	6.1	6.8
**Mean (absolute values)**				**14**		**43**			

#### 3.4.1 Data under condition of euglycemic resting state

Figure 4 compares our model predictions with experimental data during resting state (normo‐insulinemia). Predicted IGC by microdialysis (yellow bars) show a good agreement with the experimental measurements. The relative difference between our predictions and the measurements appear in Table 2. The mean relative difference between the measurements and our predictions across the five conditions is equal to 6.6% and 35%, taking and without taking into account the predicted influence of the microdialysis probe on the interstitial concentration, respectively.

#### 3.4.2 Data adapted from Gudbjörnsdottir et al. (2003)

Figure 5 shows interstitial concentrations measured by Gudbjörnsdóttir et al. (2003), under different conditions involving oral glucose tolerance test and hyperinsulinemic clamp (red bars). Conditions “(0)” refers to baseline while states “(1)” and “(2)” refers to conditions of modified physiology. Predicted IGC by microdialysis show an excellent agreement with the experimental measurements under conditions with moderate glucose metabolism or shortly after the experiment onset (OGTT(0), OGTT(1), HI(0)‐one‐step clamp and HI(1)‐two‐step‐clamp), while there is a tendency for our model to underestimate IGC measured by microdialysis under conditions showing more elevated metabolism or after a longer period of altered physiology (conditions OGTT(2), HI(0)‐one‐step clamp and HI(0)‐two‐step‐clamp). Comparing our predicted $C_{i,eff}$ with IGCs reported in other studies involving microdialysis confirms this tendency (data not shown). We discuss possible causes of this discrepancy in the section *Conditions showing higher increase in glucose uptake* in the discussion. The relative difference between our predictions and the measurements appear in Table 3. The mean relative difference between the measurements and our predictions across the seven conditions is equal to 43% and 14%, taking and without taking into account the predicted influence of the microdialysis probe on the interstitial concentration, respectively.

The corresponding reported and predicted PS products in the different conditions are shown in Table 3. For the study applying hyperinsulinemic clamp, we determined the predicted PS product when considering the effect of the microdialysis probe on glucose concentration measurements (PS_m_), and when not ('true' PS). To take into account the effect of the microdialysis probe on the glucose concentration in the PS product calculation, we used the predicted effective concentration $C_{i,eff}$, inferred from the predicted concentration $C_{i,0}$. The predicted PS product as measured with microdialysis, PS_m_, is therefore derived as:

(31) $${\text{PS}}_{m}=‐F\cdot \ln\left(\frac{C_{V}‐C_{i,eff}}{C_{A}‐C_{i,eff}}\right)=‐F\cdot \ln\left(\frac{C_{V}‐f\left(C_{i,0}\right)}{C_{A}‐f\left(C_{i,0}\right)}\right)$$

where $C_{i,eff}$ has been inferred from $C_{i,0}$ according to the function *f*, represented graphically in Figure 2 (blue curve) and Figure 3. Our model predicts that PS_m_ sharply increases between baseline and conditions with higher metabolism. Applying our model to the oral glucose tolerance test (OGTT) condition, we predict a 1.7‐fold increase in PS_m_, which is in good agreement with the 2.2‐fold increase reported by the authors. Applying our model to their hyperinsulinemic clamp condition (one‐step clamp), our model predicts a 7‐fold increase in PS_m_, which agrees reasonably well with the 11‐fold increase reported by the authors in their study. In contrast, the 'true' PS product predicted for the hyperinsulinemic clamp condition (one‐ and two‐step clamp), without taking the effect of microdialysis into account decreased to a moderate extent.

### 3.5 Determining the characteristic time to reach steady‐state IGC

To examine the rate at which IGC approaches steady‐state, we introduced time‐dependent models by including the time‐derivative $\partial C_{i}\left(r,t\right)/\partial t$ in the right hand side of Equations (15) and (27), respectively.

**FIGURE 6:**
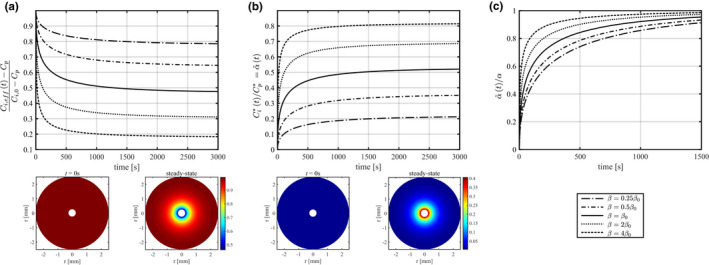
A: Interstitial glucose concentration at the immediate vicinity of the microdialysis probe, as a function of time after the glucose removal onset. B: Glucose tracer concentration at the vicinity of the probe as a function of time after adding glucose tracer to the perfusate. The concentration is normalized to the glucose tracer concentration in the perfusate $C_{p}^{*}$. As discussed in the main text, this quantity is equal to $\tilde{\alpha }\left(t\right)$, as defined in Equation (19). C: Time evolution of $\tilde{\alpha }\left(t\right)$ normalized to its steady‐state value α. This quantity can be inferred from panels A (showing $1‐\tilde{\alpha }\left(t\right)$), and B respectively. Predictions for different $\beta =\frac{R\cdot f}{S_{\mathrm{probe}}}$ values in the range [$0.25\beta_{0}$, $4\beta_{0}$] have been made, as indicated in the figure, with $\beta_{0}$ corresponding to parameters indicated in Table 1. Two‐dimensional plots at the bottom end of panels A and B show concentration profiles around the microdialysis probe at time *t* = 0 s, and at steady‐state after glucose removal by microdialysis (A) or diffusion of glucose tracer around the microdialysis probe (B) for $\beta =\beta_{0}$

Figure 6 illustrates the time needed to reach steady‐state IGC for different values of β in the range [0.25 $\beta_{0}$, 4 $\beta_{0}$]. Figure 6a presents the concentration at the vicinity of the probe as a function of the time after glucose removal onset. As glucose is removed by the probe, the concentration at the vicinity of the probe $C_{i,eff}\left(t\right)$ decreases from $C_{i,0}$ to $C_{i,eff,\text{steady ‐ state}}$ (referred to as $C_{i,eff}$, without the time dependence, in the rest of this study), with a characteristic time equal to 120 s for $\beta =\beta_{0}=6.63\cdot 10^{‐7}m\cdot s^{‐1}$, and equal to 270 and 24 s, for $\beta =0.25\beta_{0}$ and $\beta =4\beta_{0}$, respectively. For $\beta =\beta_{0}$ (resp. $\beta =0.25\beta_{0}$ and $\beta =4\beta_{0}$), interstitial concentrations are therefore predicted to reach steady‐state in 10 minutes (resp. 22 and 2 min) after glucose removal onset. Note that because interstitial concentrations of glucose and glucose tracer are essentially governed by same equations, the curves in the three figure panels are characterized by similar dynamics and characteristic times. Figure 6b presents the increasing glucose concentration around the probe as tracer is added to the perfusate. At steady‐state, the tracer concentration difference is predicted to substantially reduce net diffusion of glucose tracer out of the probe, and hence its extraction, leading to an underestimation of glucose recovery when using the internal reference calibration technique. Figure 6c shows the quantity $\tilde{\alpha }\left(t\right)=\frac{C_{i,0}‐C_{i,eff}\left(t\right)}{C_{i,0}‐C_{p}}$, normalized to its steady‐state value, as a function of time. This representation allows for a comparison between the rates at which $\tilde{\alpha }\left(t\right)$ approaches steady‐state depending on the used probe parameters.

## 4 DISCUSSION

### 4.1 Interstitial and cross‐endothelial glucose concentration gradients in resting, basal state, and corrections of microdialysis measurements

We derived a glucose diffusion model, whose relations (1) through (8) can be applied directly to experimental data to infer interstitial concentrations $C_{i,0}$ from tissue blood flow and tissue metabolism, respectively. The second model we developed in this study can be applied to correct for microdialysis‐related underestimation by solving Equation (15) with boundary conditions (16). By doing so, $C_{i,eff}$ can be inferred from $C_{i,0}$, as shown in Figures 2 and 3. Alternatively, a quick estimate of $C_{i,0}$ and $C_{i,eff}$ can be obtained from relation (30), and graphically from Figure 3, respectively.

This approach can in principle be applied to correct for microdialysis measurements of interstitial concentrations of glucose and other solutes, in muscles as well as in other organs and tissue types, such as neurotransmitters in the brain (Chefer et al., 2009), insulin, lactate and glycerol in muscle and adipose tissues (Rosdahl et al., 1998), and also exogenous compounds (Menacherry et al., 1992). To yield accurate results for other solutes, the source term γ in Equation (10) must be adapted to reflect their transport properties, which may be different from that of glucose. Parameters $D_{i}$ and $V_{i}$ must also be set according to solute and tissue type, respectively. For the model predictions to be as accurate as possible, microdialysis parameters must be calibrated accordingly. These parameters include: probe relative recovery, probe surface, probe flow rate, and perfusate solute concentrations.

One finding in this study is that, in resting muscle, under basal plasma insulin concentrations, glucose concentration gradients across the endothelium and within the interstitial fluid, respectively, are small compared to normal plasma glucose concentrations.

A second finding is that interstitial glucose concentrations are underestimated substantially due to the continuous removal of fluid during microdialysis measurements. This was expected given the rate at which microdialysis probe removes glucose. The results presented in Figures 1, 2, 3, 4, 5 show the predicted measured interstitial concentrations assuming no bias in the calibration of the relative recovery *R*, that is, $\tilde{\alpha }=0$. In that sense, they constitute a worst case scenario, in which the combined bias (bias related to glucose removal during the measurement and during the calibration of *R*) is maximal, as the two sources of bias tend to compensate – see Equation (25). Our model predicts that $C_{i,m}$ is in the range [$C_{i,eff}$,$C_{i,0}$] depending on the experimental protocol and on the calibration method employed. For example, calibrating *R* with a method allowing for quick measurements, such as the internal reference calibration technique, would lead to a measured IGC concentration close to $C_{i,eff}$. On the contrary, employing the no‐net‐flux calibration method and waiting for IGC to reach steady‐state for each dialysate measurement would lead to a measured concentration $C_{i,m}$ closer to $C_{i,0}$.

Several factors, determined during and after the calibration of the probe's recovery, thus contribute to the measurement bias $C_{i,0}‐C_{i,m}$. The most influent are the probe parameters (which determines the value of $\beta =\frac{R\cdot f}{S}$), glucose perfusate concentration $C_{p}$, as well as the value of $\tilde{\alpha }\left(t\right)$, that depends on the closeness of IGC to the steady‐state regime during the calibration of *R*. These factors are likely to vary substantially between studies and may contribute to the large IGC variance observed in the literature. Accordingly, the probes with large outer surface areas used in McConell et al. (2020), or the large glucose concentration in the perfusate used in Müller et al. (1996), limits the glucose flux into the probe, leading to smaller bias than in other studies that we applied our model to (see Figure 4). Importantly, our model predicts that incorporating glucose in perfusate in concentrations commonly reported (1.5–2 mM) does not prevent the measured IGC from being substantially biased. Note that the relative recovery has been reported to vary with the temperature of the medium and the physiological conditions under which the measurement has been performed (MacLean et al., 1999). These variations may be attributed to the dependence of the interstitial diffusion coefficient on the temperature and the tissue tortuosity coefficient. Although the recovery value is assumed to be constant throughout most studies, it could therefore vary substantially across conditions of a given study, and lead to additional glucose concentration inaccuracies (MacLean et al., 1999). This intra‐study variability is well illustrated by conditions HI(1)‐one‐step clamp and HI(1)‐two‐step clamp in Figure 5 and Table 3, for which reported IGC varies by more than 50%, despite similar experimental protocol and rate of insulin infusion (Gudbjörnsdóttir et al., 2003). As discussed in the section *Limitations of the study* below, this study focuses on bias related to the removal of glucose by the microdialysis probe, while other methodological issues with microdialysis were ignored. While some inter‐study IGC variability therefore remains after correcting for this bias, we expect average IGC estimates to be *higher* after correction.

Resting muscle IGC reported in the literature range from 50 to 100% of blood plasma levels, but our first model predicts this concentration to be only 6% lower than in blood plasma. Meanwhile, our second model predicts that microdialysis underestimates interstitial glucose levels by up to 31%. Accordingly, we predict that IGC appears to be 35% lower than plasma concentration when measured with microdialysis, which is in agreement with reported values in the literature. These predictions suggest that the variability of the reported measurements between studies is at least partly attributable to the influence of microdialysis on interstitial concentrations.

Many authors have concluded that glucose transport across the endothelium is a limiting step, especially during rest, based solely on the presence of a measured concentration gradient between plasma and interstitial fluid (Gudbjörnsdóttir et al., 2003, 2005; Holmäng et al., 1998; MacLean et al., 1999; Müller et al., 1996; Regittnig et al., 2003). Rather than limiting glucose transport into muscle cells during rest, our findings suggest that the endothelium permeability permits sufficient interstitial glucose concentration for cellular glucose uptake.

### 4.2 Conditions showing higher increase in glucose uptake

As muscle metabolism increases, for example as a consequence of increased plasma insulin concentration, our model predicts that cross‐endothelial concentration gradient, as well as gradients within the interstitial fluid, increase by the same relative amount. Under these conditions of increased metabolism, for example under conditions of hyperinsulinemia (Figure 5 and Table 3), our model shows a tendency to underestimate reported IGCs. These observations suggest that the endothelium diffusion coefficient for glucose *D_w_*, and hence its apparent permeability, which we assumed to be constant in our study, must increase under conditions of increased metabolism, as noted already 50 years ago by Renkin and Pappenheimer (Renkin, 1977). Indeed, the assumption that glucose is transported across the endothelium by simple diffusion, with a constant diffusion coefficient, is oversimplifying: The mechanisms underlying glucose transport across the endothelium in peripheral tissues are complex (Yazdani et al., 2019), and the extent to which paracellular and transcellular transfer contribute to glucose transport has yet to be elucidated (Yazdani et al., 2019). Different mechanisms have been hypothesized to increase endothelial permeability during instances of elevated metabolism. These mechanisms include increased micropinocytotic vesicles density (Østerby et al., 1978), or increased shear stress on the glycocalyx (Shibata & Kamiya, 1992; Tarbell, 2010), that constitutes an unstirred layer at the luminal surface on the capillary endothelium, owing to increased blood flow as often observed during instances of elevated metabolism.

### 4.3 PS product determined by our model

We computed the apparent PS product as we applied our model to experimental data. Our model predicts the ‘true’ PS product to show little variations between conditions owing to the assumption of constant capillary permeability and surface exchange. These predictions are in contrast to PS products reported in Gudbjörnsdóttir et al. (2003), which increase significantly in conditions with larger tissue glucose uptake, such as OGTT or hyperinsulinemia, compared to the basal conditions.

We then evaluated PS_m_, the PS product that would be inferred from plasma glucose concentrations, and IGC as measured with microdialysis, taking the modeled measurement bias into account. Interestingly, according to our model, underestimating IGC leads to underestimating PS. Moreover, as discussed above, the measurement bias associated with microdialysis affects interstitial concentrations relatively more during resting state than during hyperinsulinemia. In the study by Gudbjörnsdóttir et al. (2003), measurement bias may therefore affect basal condition PS estimates more than those recorded during hyperinsulinemia. As a result, the increase in permeability between these two conditions may be less than reported in their study.

### 4.4 Limitations of the study

In this study, we only address one out of several, methodological factors that might affect microdialysis measurements and hence contribute to the variability observed between IGC measurements. For instance, tissue damage related to the insertion of the probe may cause IGC to increase during the first 1–1.5 h following the probe insertion, possibly as a consequence of glycogen breakdown within the injured muscle tissue. Some experimenters, therefore, wait for a similar time period (Rosendal et al., 2004; Vissing et al., 2001) to allow cell membranes to reseal, so that measurements are indeed conducted on interstitial fluid.

In this study, we focused exclusively on resting muscle, during normo‐ and hyperinsulinemia, respectively. The effects of muscle contraction on the effective diffusion coefficient in the endothelium or in the interstitial fluid, due for instance to variations in temperature or tortuosity are complex and incompletely understood. When such data become available, these effects can readily be taken into account in our model by updating the effective diffusion coefficients values $D_{w}$, and $D_{i}$ accordingly, allowing the model to be applied under other conditions.

## 5 CONCLUSION

In conclusion, using a diffusion model for glucose in skeletal muscle capillaries, we show that the concentration gradients between plasma and the interstitial fluid, as well as within the interstitial fluid, are predicted to be small and negligible, respectively, during rest. The gradient across the endothelium is predicted to increase substantially as metabolism increases. An increase in endothelial permeability accompanying hyperinsulinemia and an increase in blood flow, further ensures adequate interstitial levels to support glucose transport into the cell. The endothelium is therefore not a barrier to glucose transport under most physiological conditions.

Several methodological limitations of microdialysis have been discussed. We have shown that this technique may underestimate interstitial concentrations. These underestimations could in turn explain the observed discrepancies between interstitial concentration values reported in the literature. The model and conceptual framework presented in this study, once validated experimentally, could be used to correct for substrate removal in micro‐dialysis based concentration measurement and guide their interpretation. The model may also serve to optimize the experimental design in the choice and calibration of probe parameters (diameter, length, perfusate flow, substrate concentration in the perfusate) to optimize the accuracy of interstitial substrate concentration measurements.

Finally, our model predicts that even a modest underestimation of interstitial glucose concentrations leads to the prediction that the apparent PS product increases with metabolism, in good agreement with reports in the literature.

## CONFLICT OF INTEREST

No conflict of interest, financial or otherwise, are declared by the authors.

## AUTHORS' CONTRIBUTION

H.A. and L.Ø. conceived and designed the study; H.A. developed the model and analyzed the data; H.A. and L.Ø. interpreted the model results and predictions; H.A. prepared the figures; H.A. and L.Ø. drafted the manuscript; H.A. and L.Ø. edited and revised the manuscript; H.A. and L.Ø. approved the final version of the manuscript.

## Data Availability

The data used in this study for the model calibration are indicated in Table 1 with the corresponding references. The data related to the model predictions, as well as the model source code, are available from the corresponding author upon request.
